# Anæsthesia, and the Use of a New Ether-Inhaler

**Published:** 1893-06

**Authors:** J. E. Waitt


THE
### International Dental Journal.

Vol. XIV. June, 1893. No. 6.

### Original Communications.[1]

### ANÆSTHESIA, AND THE USE OF A NEW ETHER-INHALER.[2]

BY J. E. WAITT, D.M.D.

Mr. President and Members of the American Academy of Dental Science,—It is with a feeling of diffidence that I come before you to-night to say a few words on a subject that ought to be familiar to each and every one of you, and yet, from personal observation and the special study of the subject for nearly ten years, I am confident that very few of us are perfectly at home in the use of ether, which, since its discovery and first introduction in 1846, has done more to alleviate the sufferings of humanity than all the other discoveries combined. It is a fitting tribute to Dr. Morton that I quote right here an abstract from an account of the "First Capital Operation under the Influence of Ether," by Daniel Dennison Slade, M.D., published in the October, 1892, *Scribner.*

Dr. Slade was present at the operation and graphically describes the scene. (Dr. W. T. G. Morton, referred to in the following article, was a Boston physician. He was the first to introduce the use of sulphuric ether, and his first experiment took place at the Massachusetts General Hospital, in October, 1846.)

---

[1] The editor and publishers are not responsible for the views of authors of papers published in this department, nor for any claim to novelty, or otherwise, that may be made by them. No papers will be received for this department that have appeared in any other journal published in the country.

[2] Read before the American Academy of Dental Science, January 4, 1893.

"As all eyes were now fixed upon the scene before them, Dr. George Hayward stepped forward and remarked that, with the advice of the other surgeons, he should allow Dr. Morton to administer an article by inhalation to the patient upon whom he was about to operate which, it was alleged, would prevent any pain being felt.

"Thereupon, Morton, a man of commanding figure and appearance, very erect, and dressed, as he usually was, in a stylish fashion peculiar to himself, consisting of a blue frock-coat with brass buttons, a large and elegant scarf which completely filled up the open front of the waistcoat, 'gaiter' trousers, etc., and bearing in his hands the instrument already described, came from an adjacent room, and advancing to the operating-table, spoke a few words of encouragement to the patient and instructed her in the method of inhaling. The curiosity on the part of all present was intense. The stillness was oppressive, broken only by the hurried respiration and occasional sob of the patient. Grouped about Morton, standing as the central figure at the head of the operating-table, were the surgical and medical officers of the institution, as also the attendants, all as intent upon the unusual scene before them as were the most untried spectators in the seats of the amphitheatre.

"In three minutes the patient was completely under the influence of the preparation, as shown by the complete muscular relaxation, the drooping eyelids, the immobile pupil, and the death-like insensibility to external impressions.

"The operation completed, and even before the removal of the patient from the room, the profound stillness and suspense which had hung over all present were broken by loud murmurs of surprise and admiration at the success which had been attained. Morton was the hero of the hour, and was regarded with feelings akin to those which might have been awakened had an angel appeared, bearing the waters from 'the Lethean streams of oblivion,' which having been administered to the suffering invalid, had produced the effects witnessed."

From that memorable Saturday, November 7, 1846, to the present day, there have been discussions as regards the best method of administering ether and the after-result upon the patient, especially after laparotomy, where it is positively necessary that the patient should recover quickly from the effects of the ether without the retching that so commonly follows.

To go over the different forms of apparatus for the administering of ether would take too long a time, but I can safely say that up to within a very short period there have been none devised but required a great amount of ether in their use and failed entirely in the three important factors of an inhaler as the medium in administering ether,—viz., rapid anæsthesia, anæsthesia easily retained, and by a minimum of ether; then a rapid recovery without its attendant evils, retching, sickness, and headache.

About five years ago I introduced an inhaler, called the Packard inhaler, to the medical and dental profession, which at that time, and even to-day, is the best form of inhaler that can be used in administering ether in the old way. I need not give a description of this, as I will pass one around, and you can see the advantages over the old towel or paper cones and that most miserable of all, the sponge.

In the new Packard inhaler which I present to you this evening you will see the result of many years' study and experimenting, and in it a means of safely, economically, and pleasantly administering ether. It involves an entirely new principle,—namely, anæsthesia with etherated air.

It comprises an ordinary face-piece, such as is in use with the gas apparatus of to-day; to that part which contains the shut-off is soldered a shoulder, for the attachment of a rubber sponge-bag, in the top of which is placed a slide-ring valve; through the side of the inhaler is passed a tube, with the inner end bent so as to enter the bag. A graduated bottle holding four ounces contains the ether. This bottle is fitted with a screw cap, which has fastened through the top two tubes, one passing to the bottom of the bottle and the other just entering the cap; each tube has a slip-joint so made that the rubber tubes which are fastened to them are securely held. An ordinary thermo-cautery bulb completes the inhaler.

Attaching the parts, as you will see, then compressing the bulb, air is driven through the ether, thereby becoming thoroughly etherated; this is then forced into the bag of the inhaler, and from thence to the patient.

This seems a complicated form, but by a practical test of but two or three inhalations you will see that it is very simple. The advantages are numerous. First, no ether comes in direct contact with the patient's face or skin, therefore all burning of the face and eyes is avoided. Secondly, as the ether vapor is wanted just so fast is it made, thereby saving all evaporation of ether and saturation of the surrounding atmosphere with its disagreeable fumes.

The results of over one hundred tabulated administrations show that the average amount of ether required to produce surgical anæsthesia was three drachms, and for each hour's consumption two ounces. The average time of inducing complete anæsthesia, five and one-half minutes; longest time, nine minutes; shortest time, two and one-half minutes.

The recovery is rapid, exceedingly so; many patients are conversing with the operator or nurse in three minutes after the inhaler is removed from the face.

Possibly a few words in regard to the best way of using the inhaler will clear away some doubts. This method I find the most satisfactory.

DIRECTIONS FOR USE.

1. Place from two to four ounces of ether, according to the estimated duration of the operation, in the bottle.

2. Place the hood over the patient's face, looking carefully to see that it fits air-tight about the chin and over the nose.

3. See that the air-valve on front of metal hood is wide open.

4. Compress the air-bulb very gently, just enough to drive a few bubbles of air through the ether.

5. At the end of the first minute close the air-valve.

6. Gradually increase the rapidity and force of the bulb compressions. One moderate compression with each inspiration is sufficient in the first stage.

7. As the patient approaches the second stage of anæsthesia, make two compressions of the bulb with each inspiration.

In from six to eight minutes the patient will be completely anæsthetized, with the consumption of from two to six drachms of ether.

During the progress of anæsthesia the air-valve can usually be kept partly open. At any time, in case of coughing, choking, or obstructed respiration, the air-valves should be partly or wholly opened.

Two and one-half ounces of ether should suffice for each hour of anæsthesia.

DON'TS.

Don't attempt to etherize a patient with the stomach full.

Don't use old ether which has stood in a warm closet or in an imperfectly-stoppered bottle.

Don't use anything but Squibb's four-ounce cases of ether.

Much of our success as anæsthetists depends upon our manner with our ether patients. We should see the patient a day or two before administering the ether, and in a quiet talk tell him of the peculiar features connected with its administration.

To be successful, circumstances must be brought under our control as much as possible, the patient in a submissive frame of mind, willing to yield entirely to us.

If a patient is frightened or roughly handled he naturally rebels, and the process is necessarily prolonged and therefore imperfect.

Children and adults of impressible natures are more easily overcome than patients of a vigorous intellectual character. Will may delay the progress of anæsthesia, and minds trained in reasoning will retain consciousness much longer than if less happily organized.

Ether should be administered three or four hours after meals, with the clothing in a loose condition at the neck and waist, and the patient in a recumbent position or nearly so.

In administering ether the eyes of the patient should be covered at the beginning of the inhalation; this precaution shuts out external observation and hastens the period of insensibility.

It is also advantageous to have the attention of the patient diverted by counting slowly one, two, three, and so on, following the lead of the administrator. The expiratory effort by so doing is followed by corresponding inspiration, which assists materially the full inhalation of ether.

Few patients can count as high as twenty-five or thirty without feeling the effect of the agent, and scarcely any one can reach sixty or seventy before becoming unconscious.

The best tests for the proper period for operating are insensibility of the eyeball to the touch, non-contraction of the pupil on the sudden exposure to light, and the general muscular relaxation.

The ether may now be withheld or pushed to any desired extent.

The unconscious occupation of the mind by counting is far better than coaxing a patient to try and go to sleep, which, under the circumstances, is very difficult.

In administering ether the pulse and the breathing should be closely watched. If the pulse be strong, the patient is doing well; if it becomes feeble, the ether should be withdrawn and the access of air permitted. When mucus collects in the mouth and throat it must be removed at once by a wet sponge or by carrying the finger back of the retracted tongue and drawing it forward.

One more word and I am done, and that is concerning the after-treatment of our patients. They should never be left alone or unwatched until they have regained consciousness, or until the respiration, circulation, and color of the skin have been fairly established.

Frequently anæsthesia is followed by prolonged sleep, particularly in young patients. This may be overcome by bathing the face in cold water.

Fortunately, however, looking at the great number of cases in which ether is given with perfect impunity, accidents are very rare; so rare, in fact, that when they do occur it is a question whether they were the result of administering the ether or from some other cause.

